# The efficacy and safety of different Janus kinase inhibitors as monotherapy in rheumatoid arthritis: A Bayesian network meta-analysis

**DOI:** 10.1371/journal.pone.0305621

**Published:** 2024-06-21

**Authors:** Bingjia Qu, Feng Zhao, Ying Song, Junyi Zhao, Yuxin Yao, Yulan Chen, Ruobing Liao, Lingyu Fu

**Affiliations:** 1 Department of Clinical Epidemiology and Evidence-based medicine, The First Hospital, China Medical University, Shenyang, China; 2 Department of Medical Record Management Center, The First Hospital, China Medical University, Shenyang, China; University of Illinois, UNITED STATES

## Abstract

**Objective:**

This study aims to evaluate the efficacy and safety of JAK inhibitors in the treatment of patients with RA.

**Methods:**

The databases CNKI, VIP, Wanfang, CBM, and PubMed, Embase, Cochrane Library and Web of Science were searched to identify relevant randomized controlled trials (RCTs), all from the time of database creation to April 2024. Screening, data extraction, and risk of bias assessment (using Review Manager-5.3 software) were independently performed by at least two authors. The network meta-analysis was conducted using R 4.1.3 software. PROSPERO registration number: CRD42022370444.

**Results:**

Thirty-three RCTs included 15,961 patients The experimental groups involved six JAK inhibitors (filgotinib, tofacitinib, decernotinib, baricitinib, upadacitinib and peficitinib) and 12 interventions (different doses of the six JAK inhibitors), and the control group involved adalimumab (ADA) and placebo. Compared with placebo, all JAK inhibitors showed a significant increase in efficacy measures (ACR20/50/70). Compared with ADA, only tofacitinib, low-dose decernotinib, and high-dose peficitinib showed a significant increase in ACR20/50/70. Decernotinib ranked first in the SUCRA ranking of ACR20/50/70. In terms of safety indicators, only those differences between low-dose filgotinib and high-dose upadacitinib, low-dose tofacitinib and high-dose upadacitinib were statistically significant. Low-dose filgotinib ranked first in the SUCRA ranking with adverse events as safety indicators. Only the efficacy and safety of tofacitinib ranked higher among different SUCRA rankings.

**Conclusion:**

Six JAK inhibitors have better efficacy than placebo. The superior efficacy of decernotinib and safety of low-dose filgotinib can be found in the SUCRA. However, there are no significant differences in safety between the different JAK inhibitors. Head-to-head trials, directly comparing one against each other, are required to provide more certain evidence.

## Introduction

Rheumatoid arthritis (RA) is an autoimmune disease of unknown etiology, which is characterized by synovitis. It is often associated with involvement of organs other than joints and positive serum rheumatoid factor test results, which can lead to joint deformity and loss of motion in severe cases. However, since the pathogenesis of RA is not clearly understood at this stage, several commonly used RA drugs are anti-rheumatic drugs that have the effect of delaying symptoms and improving the disease. For example, small molecule drugs such as methotrexate and glucocorticoids are more likely to produce toxic side effects such as hypertension, heart disease, obesity and other conditions during the long-term treatment process, and patient compliance is also poor [1]. Not only that, it one-third of patients are unable to tolerate methotrexate due to its adverse side effects or inadequate response, which leads to its discontinuation [2]. Even with the application of biologics, an increasing number of patients have become resistant thereto, and only 20%-25% of patients with RA have achieved complete relief of their symptoms [1].

Therefore, it is necessary to explore new drugs for the treatment of RA, the Janus kinase (JAK) inhibitor class, where different JAK inhibitors can act by inhibiting different pathways. Upadacitinib and filgotinib can act through the inhibition of JAK1, but the high selectivity of filgotinib for JAK1 can reduce the range of adverse effects caused by inhibiting of JAK2 and therefore would be well tolerated and safe. Both peficitinib and decernotinib are selective JAK3 inhibitors. The former was launched in 2016 and approved in Japan in March 2019 for the treatment of RA patients who have a poor response to traditional DMARDs drugs. The latter has been the subject of fewer existing studies, but one study showed that in RA patients, after 12 weeks of treatment, decernotinib had a therapeutic effect compared to placebo [3]. Tofacitinib and baricitinib can inhibit two pathways simultaneously, JAK1 and JAK3 and JAK1 and JAK2 [4,5].

Several randomized controlled trials (RCTs) in different countries have evaluated the efficacy of tofacitinib, baricitinib, peficitinib, upadacitinib, filgotinib and decernotinib as monotherapy in patients with RA by comparing them with placebo [3,6–10], in which different JAK inhibitors showed better efficacy compared to placebo, but because the data from head-to-head studies comparing various JAK inhibitors at different doses are sparse, it is necessary to compare the efficacy of different JAK inhibitors at different doses in combination with evidence from RCTs of different treatments. Thie present study used a Bayesian reticulation meta-analysis to examine the relative efficacy and safety of various JAK inhibitors in RA patients.

## Patients and methods

This systematic evaluation and meta-analysis was reported in accordance with PRISMA-NMA reporting norms [11], under PROSPERO registration number: CRD42022370444.

### Literature search strategy

A search of CNKI, VIP, Wanfang, CBM, and Pubmed, Embase, Cochrane Library and Web of Science databases was conducted to collect RCTs on JAK inhibitors for RA, and the search time frame was from creation to April 2024 in all cases. To supplement access to pertinent literature, references to the contained literature were retroactively included. The search was carried out using a combination of subject terms and free words. English search phrases were used, including "rheumatoid arthritis", "JAK kinase inhibitors", "tofacitinib ", "baricitinib", "upadacitinib", "filgotinib", "decernotinib". "decernotinib", "peficitinib", etc. Two researchers independently searched for eligible articles. In case of disagreement, a third researcher was called to make the decision.

### Eligibility and participants

Study subject participants aged 18 years or older and diagnosed with RA who meet the American College of Rheumatology (ACR) 1987 revised criteria or the new rheumatoid classification criteria jointly proposed by the ACR and the European League Against Rheumatism (ACR/EULAR) in 2010, regardless of the patient’s disease duration, gender, or race were selected.

### Interventions, comparators, and outcome measures

Interventions with JAK inhibitors include: different doses of filgotinib (100 mg QD-low-dose and 200 mg QD-high-dose), different doses of tofacitinib (5 mg BID-low-dose and 10 mg BID-high-dose), different doses of decernotinib (100 mg BID-low-dose and 150 mg BID-high-dose), different doses of baricitinib (2 mg QD-low-dose and 4 mg QD-high-dose), different doses of upadacitinib (15 mg QD-low-dose and 30 mg QD-high-dose), and different doses of peficitinib (100 mg QD-low-dose and 150 mg QD-high-dose).

The following comparators were included: adalimumab (ADA) 40 mg Q2W and placebo.

Indicators of outcome: the number of patients who achieve ACR20 (ACR20%) after drug administration is a primary outcome indicator; secondary outcome indicators include the number of patients who achieve ACR50 (ACR50%), ACR70 (ACR70%) and adverse events (adverse events are defined as any unfavorable medical occurrences following the administration of a subject’s medication, which may present as symptoms, signs, diseases, or abnormal laboratory findings, such as headache, nausea, nasopharyngitis, infection, *etc*.).

### Study design

The current study only included RCTs and excluded studies in which the following situations occured: 1) duplicate literature by the same investigator; 2) animal studies, reviews and systematic evaluations, and case reports; 3) literature with incomplete outcome indicators and low quality was not available (after study quality evaluation, the results showed that high-risk or unreasonable research design and conduct could be deemed to have been low-quality research).

### Data extraction

Independently reviewing the literature was undertaken by two researchers, who extracted information and cross-referenced, it before consulting a third person to aid in decision-making in the event of conflict, and contacting the authors to supplement any lacunae as far as much as possible. After discarding any obviously unrelated literature, the title and abstract of the paper were read to screen the literature. A second reading of the literature was then performed to ascertain whether or not it should be included in the final analysis. The data extraction mainly included: 1) basic information of the included studies: title, trial name, first author, and year of publication; 2) baseline characteristics of the study population: number of trial participants, gender, age, duration of disease, DAS28-ESR and DAS28-CRP; 3) specific details of the interventions, follow-up time, background; 4) key elements of risk of bias; 5) outcome indicators and outcome measures of interest: ACR20, ACR50, ACR70 and adverse events.

### Study quality

Two review authors independently extracted data from the included trials. Data were extracted and collated using a standardized, agreed upon, data extraction form. Data collected included: one review author (SA) transferred the data into the Review Manager 5 file (RevMan 2014). A second review author double- checked that the data were entered correctly, by comparing the data presented in the systematic review with the data extraction form, and spot-checked study characteristics for accuracy against the trial report. The risk of bias of the included RCTs was evaluated by two researchers according to the risk of bias evaluation tool for RCTs in the Cochrane Handbook [12].

### Data analysis

Review Manager-5.3, JAGS, and R 4.1.3 software and its network, BUGSnet package were adopted to perform the network Meta-analysis and to draw the evidence network diagram for each effect indicator and the funnel diagram for assessing publication bias. The SUCRA was calculated for various interventions and plotted for ranking to derive the probability of the best intervention for the outcome indicator. All the outcome indicators in this study were dichotomous variable information, and the relative risk ratio (RR) was used as two kinds of indicators, and 95% confidence interval (95% CI) was given. Funnel plots were used to evaluate likelihood of the presence/absence of a small sample effect or publication bias.

The posterior density for unknowable variables was calculated using the Markov Chain Monte Carlo method. The most appropriate and conservative method of analysis to take variations between RCTs into account was the random-effects model. With separate initial values that were picked at random for convergence, two Markov chains ran concurrently. For each of the two sets of beginning values, 50,000 simulations were produced, with the first 10,000 simulations being discarded as the burn-in period [13,14].

Sensitivity analyses for efficacy indicators (ACR20/50/70) and safety indicators (AE) were performed. The deviance information criterion (DIC) is usually used to compare the goodness of fit between fixed-effects models and random-effects models, and the DIC difference is also used as the result of sensitivity analysis. The DIC of consistency and inconsistency models can also be employed to evaluate the consistency of the model. The smaller the DIC value, the better the model, and it is generally considered that DIC value greater than or equal to five units denotes a difference between the two models.

## Results

### Search results

**Fig 1:**
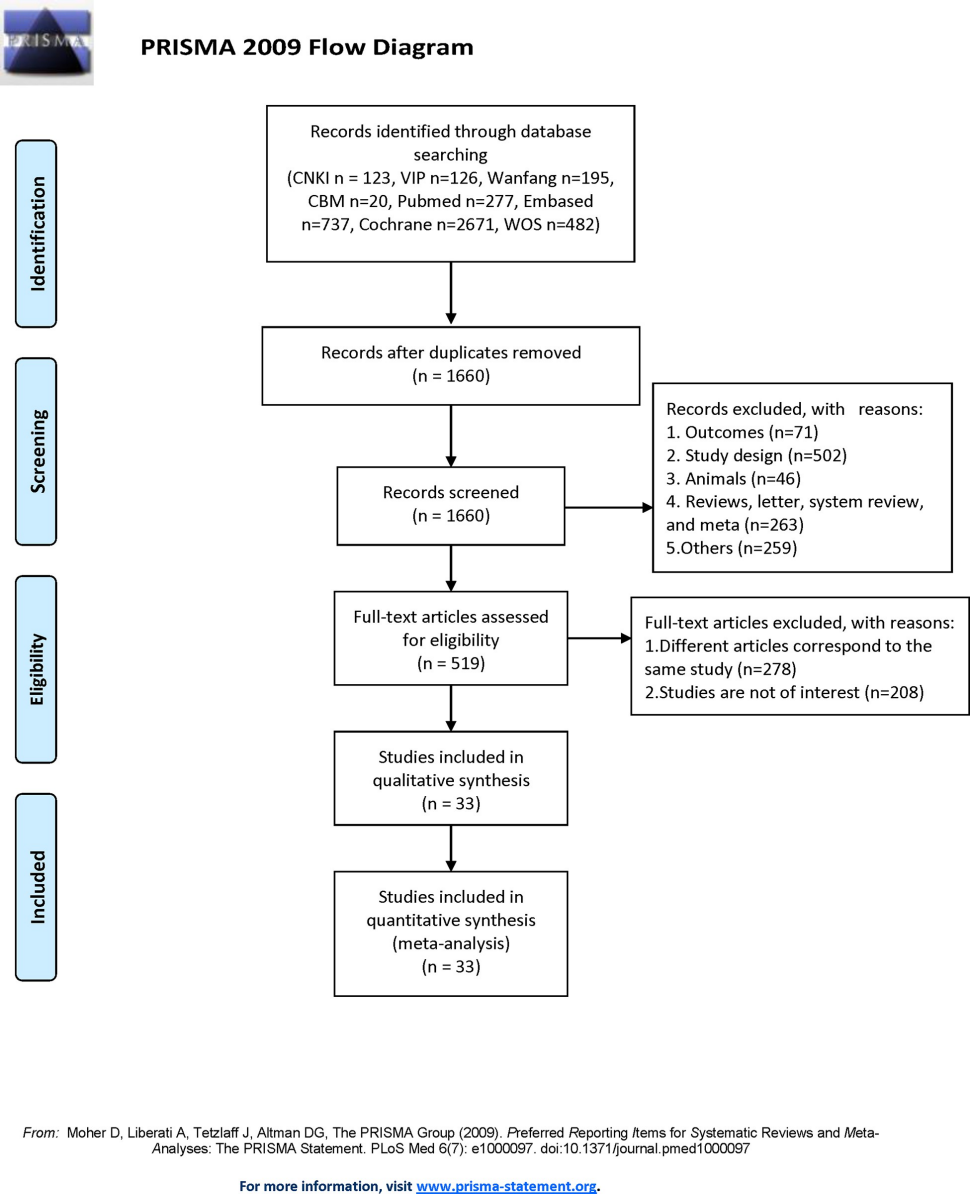
Flow chart of the selection process of articles.

4,631 articles were identified after searching the database. 2,971 duplicates were eliminated throughout the screening process. 1,141 articles were disqualified after titles and abstracts were examined. Following that, 486 articles were removed after reading 519 full-text articles. Consequently, 33 pieces of literature and research in all were applied in the analysis (**Fig 1**).

### Characteristics of included studies

**Fig 2:**
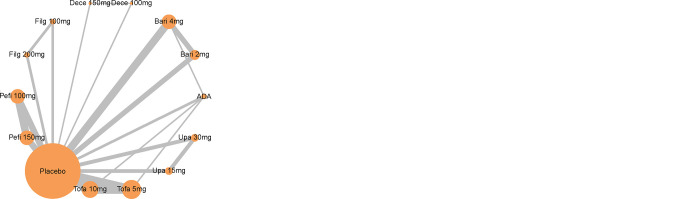
The selection of RCTs included in the NMA. (a) ACR 20; (b) ACR 50/70; (c) adverse events.

33 RCTs (15,961 participants) and different doses of six types of JAK inhibitors were included (**Fig 2**). The basic characteristics of the included studies are presented in **S1 Table**. A total of 31 RCTs [3,5,7–10,15–39] involved in ACR 20/50/70, 30 RCTs [3,5,7–10,15–38] involved in ACR 50/70, and 26 RCTs [3,5,7,8,16,18–30,32,33,35–37,39–41] reported the occurrence of adverse events involving 13 interventions which included: different doses of filgotinib, tofacitinib, decernotinib, upadacitinib, peficitinib and baricitinib, and the control group included: ADA and placebo.

### Risk of bias

The studies included in this research were of moderate quality. All studies generated randomization sequences. 14 (42%) concealed allocation. 31 research papers (94%) reported blinding of participants and personnel. 2 (6%) blinded the outcome assessors. 26 studies (79%) indicated that the outcome indicators were well-completed. The risk of bias of all included studies is shown in **S1 Fig**.

### Efficacy

**Fig 3:**
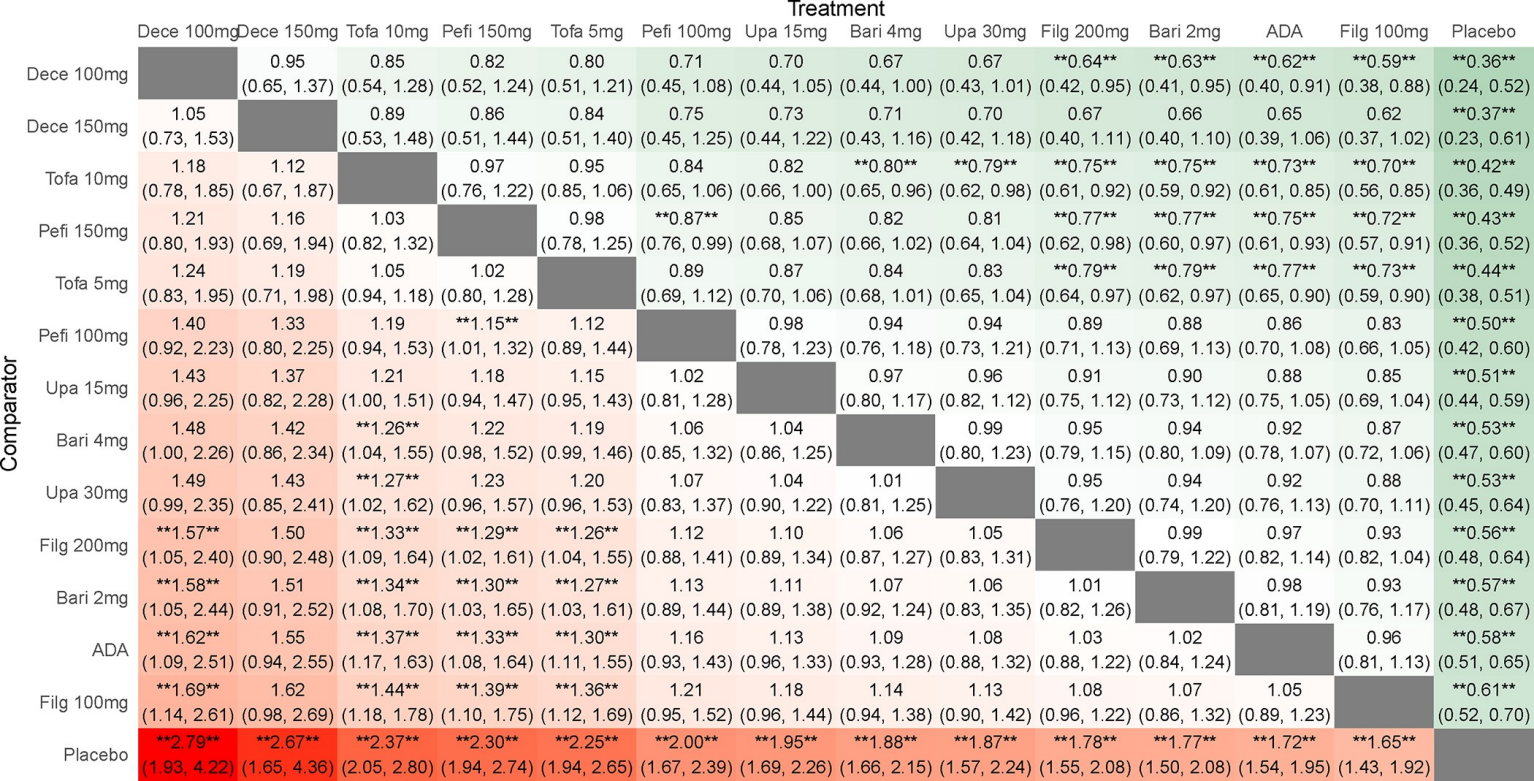
Results of a network meta-analysis of JAK inhibitors for ACR20 in RA. Data are relative risks and their related 95% credible intervals. *, statistically significant differences between the two interventions.

**ACR20.** By comparing the DIC of consistency model and inconsistency model, the DIC of consistent model is found to be smaller. When compared with placebo, the efficacy indicator ACR20 was significantly increased for all JAK inhibitors (*P* < 0.05) and when compared with ADA, ACR20 was significantly increased for both high and low-dose tofacitinib (RR 1.37, 95%CI 1.17–1.63; RR 1.30, 95%CI 1.11–1.55), low-dose decernotinib (RR 1.62, 95%CI 1.09–2.51) and high-dose peficitinib (RR 1.33, 95%CI 1.08–1.64). Indirect comparison revealed that the efficacy of low-dose decernotinib (RR 1.57, 95%CI 1.05–2.40; RR 1.69, 95%CI 1.14–2.61), different doses of tofacitinib (RR 1.33, 95%CI 1.09–1.64; RR 1.44, 95%CI 1.18–1.78; RR 1.26, 95%CI 1.04–1.55; RR 1.36, 95%CI 1.12–1.69), and high-dose peficitinib (RR 1.29, 95%CI 1.02–1.61; RR 1.39, 95%CI 1.10–1.75), was superior to that of different doses of filgotinib with statistically significant differences therein. The efficacy of low-dose decernotinib, high-dose peficitinib and different doses of tofacitinib was superior to that of low-dose baricitinib (RR 1.58 95%CI 1.05–2.44; RR 1.30 95%CI 1.03–1.65; RR 1.34, 95%CI 1.08–1.70; RR 1.27 95%CI 1.03–1.61), with statistically significant differences. In addition, the efficacy of high-dose tofacitinib was significantly better than that of high-dose baricitinib and upadacitinib (RR 1.26 95%CI 1.04–1.55; RR 1.27, 95%CI 1.02–1.62), and the efficacy of high-dose peficitinib was significantly better than that of its low-dose (RR 1.15 95%CI 1.01–1.32), with statistically significant differences (**Fig 3**). The SUCRA of ACR20 was in the order of low-dose decernotinib > high-dose decernotinib > high-dose tofacitinib > high-dose peficitinib > low-dose tofacitinib > low-dose peficitinib > low-dose upadacitinib > high-dose baricitinib > high-dose upadacitinib > high-dose filgotinib > low-dose baricitinib > ADA > low-dose filgotinib > placebo, suggesting low-dose decernotinib was most effective in RA patients with ACR20 as the efficacy evaluation index (**Table 1**).

**Table 1:** Efficacy index SUCRA scores and ranking.

Treatment	SUCRA	Treatment	SUCRA	Treatment	SUCRA
ACR20		ACR50		ACR70	
low-dose decernotinib	0.912	high-dose decernotinib	0.951	high-dose decernotinib	0.798
high-dose decernotinib	0.842	low-dose decernotinib	0.857	high-dose upadacitinib	0.796
high-dose tofacitinib	0.834	high-dose tofacitinib	0.757	low-dose decernotinib	0.721
high-dose peficitinib	0.786	high-dose peficitinib	0.705	high-dose peficitinib	0.705
low-dose tofacitinib	0.742	low-dose tofacitinib	0.646	high-dose tofacitinib	0.672
low-dose peficitinib	0.534	high-dose upadacitinib	0.636	high-dose baricitinib	0.602
low-dose upadacitinib	0.511	low-dose upadacitinib	0.611	low-dose upadacitinib	0.591
high-dose baricitinib	0.438	high-dose baricitinib	0.473	low-dose baricitinib	0.557
high-dose upadacitinib	0.407	high-dose filgotinib	0.420	high-dose filgotinib	0.468
high-dose filgotinib	0.320	low-dose peficitinib	0.369	low-dose tofacitinib	0.411
low-dose baricitinib	0.289	low-dose baricitinib	0.255	low-dose peficitinib	0.301
ADA	0.230	low-dose filgotinib	0.183	low-dose filgotinib	0.267
low-dose filgotinib	0.154	ADA	0.141	ADA	0.111
placebo	0	placebo	0	placebo	0

SUCRA, surface under the cumulative ranking curve; ADA, adalimumab; filgotinib 100 mg QD, low-dose filgotinib; filgotinib 200 mg QD, high-dose filgotinib; tofacitinib 5 mg BID, low-dose tofacitinib; tofacitinib 10 mg BID, high-dose tofacitinib; decernotinib 100 mg BID, low-dose decernotinib; decernotinib150 mg BID, high-dose decernotinib; baricitinib 2 mg QD, low-dose baricitinib; baricitinib 4 mg QD, high-dose baricitinib; upadacitinib 15 mg QD, low-dose upadacitinib; upadacitinib 30 mg QD, high-dose upadacitinib; peficitinib 100 mg QD, low-dose peficitinib; peficitinib 150 mg QD, high-dose peficitinib.

#### ACR50

**Fig 4:**
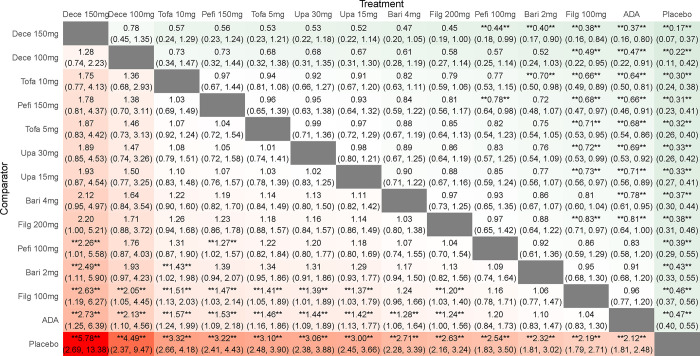
Results of a network meta-analysis of JAK inhibitors for ACR50 in RA. Data are relative risks and their related 95% credible intervals. *, statistically significant differences between the two interventions.

By comparing the DIC of consistency model and inconsistency model, the DIC of consistent model was found to be smaller. When compared with placebo, the efficacy indicator ACR50 was significantly increased for all JAK inhibitors (*P* < 0.05) and when compared with ADA, ACR50 was significantly increased for tofacitinib (RR 1.57, 95%CI 1.24–1.99; RR 1.46, 95%CI 1.16–1.86), decernotinib (RR 2.73 95%CI 1.25–6.39; RR 2.13, 95%CI 1.10–4.56), upadacitinib (RR 1.44 95%CI 1.09–1.89; RR 1.42, 95%CI 1.13–1.77), high-dose baricitinib (RR 1.28 95%CI 1.06–1.64), high-dose filgotinib (RR 1.24 95%CI 1.00–1.56), and high-dose peficitinib (RR 1.53, 95%CI 1.09–2.18). Indirect comparison indicated that the efficacy of decernotinib (RR 2.63, 95%CI 1.19–6.27; RR 2.05, 95%CI 1.05–4.45), tofacitinib (RR 2.63, 95%CI 1.19–6.27; RR 2.05, 95%CI 1.05–4.45), upadacitinib (RR 1.39, 95%CI 1.01–1.89; RR 1.37, 95%CI 1.03–1.79), high-dose peficitinib (RR 1.47, 95%CI 1.03–2.41), and high-dose filgotinib (RR 1.20, 95%CI 1.03–1.40) was superior to that of low-dose filgotinib, with statistically significant differences therein; the efficacy of high-dose decernotinib was superior to that of low-dose peficitinib (RR 2.26, 95%CI 1.01–5.58) and low-dose baricitinib (RR 2.49, 95%CI 1.11–5.90), with statistically significant differences therein. In addition, the efficacy of high-dose tofacitinib was significantly better than that of low-dose baricitinib (RR 1.43, 95%CI 1.02–1.98). Low-dose peficitinib was better than low-dose peficitinib (RR 1.27, 95%CI 1.02–1.57), with statistically significant differences (**Fig 4**). The SUCRA of ACR50 was such that high-dose decernotinib > low-dose decernotinib > high-dose tofacitinib > high-dose peficitinib > low-dose tofacitinib > high-dose upadacitinib > low-dose upadacitinib > high-dose baricitinib > high-dose filgotinib > low-dose peficitinib > low-dose baricitinib > low-dose filgotinib > ADA > placebo. The result suggested that high-dose decernotinib has the most prominent efficacy in RA patients in terms of ACR50 as the efficacy evaluation index (**Table 1**).

#### ACR70

**Fig 5:**
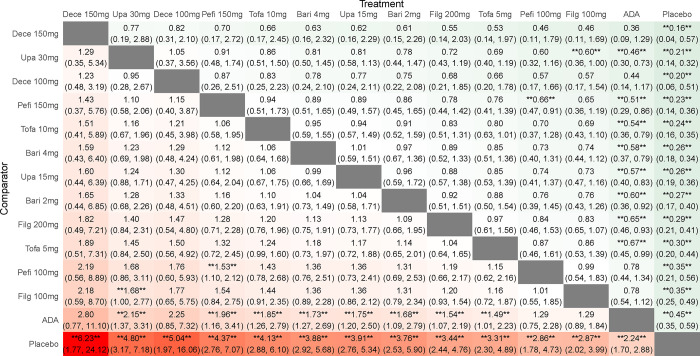
Results of a network meta-analysis of JAK inhibitors for ACR70 in RA. Data are relative risks and their related 95% credible intervals. *, statistically significant differences between the two interventions.

By comparing the DIC of consistency model and inconsistency model, the DIC of consistent model is shown to be smaller. When compared with placebo, the efficacy indicator ACR70 was significantly increased for all JAK inhibitors (*P* < 0.05) and when compared with ADA, ACR70 was significantly increased for high-dose filgotinib (RR 1.54, 95%CI 1.07–2.19), tofacitinib (RR 1.85,95%CI 1.26–2.79; RR 1.49, 95%CI 1.01–2.23), baricitinib (RR 1.73 95%CI 1.27–2.69; RR 1.68, 95%CI 1.09–2.79), upadacitinib (RR 2.15, 95%CI 1.37–3.31; RR 1.75, 95%CI 1.20–2.50), and high-dose peficitinib (RR 1.96, 95%CI 1.16–3.41). No statistically significant difference was observed regarding the ACR70 among the evaluated treatments, except between high-dose peficitinib and low-dose peficitinib (RR 1.53, 95%CI 1.10–2.21), between high-dose upadacitinib and low-dose filgotinib (RR 1.68, 95%CI 1.00–2.77) (**Fig 5**). The SUCRA of ACR70 was such that high-dose decernotinib > high-dose upadacitinib > low-dose decernotinib > high-dose peficitinib > high-dose tofacitinib > high-dose baricitinib > low-dose upadacitinib > low-dose baricitinib > high-dose filgotinib > low-dose tofacitinib > low-dose peficitinib > low-dose filgotinib > ADA > placebo. The finding shows that high-dose decernotinib has the most prominent efficacy in ACR70 (**Table 1**).

### Safety

#### Adverse events

**Fig 6:**
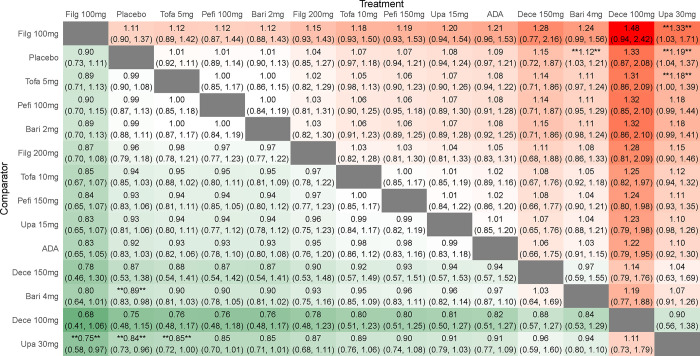
Results of a network meta-analysis of JAK inhibitors for adverse events in RA. Data are relative risks and their related 95% credible intervals. *, statistically significant differences between the two interventions.

By comparing the DIC of consistency model and inconsistency model, the DIC of consistent model was found to be smaller. When compared with placebo, except high-dose baricitinib and high-dose upadacitinib (RR 0.89 95%CI 0.83–0.98; RR 0.84, 95%CI 0.73–0.96), other drugs with different doses were as safe as the placebo. When compared with each other, the safety of low-dose tofacitinib and low-dose filgotinib was better than that of high-dose upadacitinib (RR 0.85, 95%CI 0.72–1.00; RR 0.75, 95%CI 0.58–0.97), indicating a statistically significant (**Fig 6**). The SUCRA of adverse events was such that low-dose filgotinib > placebo > low-dose tofacitinib > low-dose peficitinib > low-dose baricitinib > high-dose filgotinib > high-dose tofacitinib > high-dose peficitinib > low-dose upadacitinib > ADA > high-dose decernotinib > high-dose baricitinib > low-dose decernotinib > high-dose upadacitinib, which suggests that low-dose filgotinib is better tolerated (**Table 2**).

**Table 2:** Safety index SUCRA scores and ranking.

Treatment	SUCRA
**AE**	
low-dose filgotinib	0.900
placebo	0.749
low-dose tofacitinib	0.697
low-dose peficitinib	0.684
low-dose baricitinib	0.683
high-dose filgotinib	0.566
high-dose tofacitinib	0.459
high-dose peficitinib	0.452
low-dose upadacitinib	0.441
ADA	0.396
high-dose decernotinib	0.373
high-dose baricitinib	0.287
low-dose decernotinib	0.161
high-dose upadacitinib	0.152

SUCRA, surface under the cumulative ranking curve; ADA, adalimumab; filgotinib 100 mg QD, low-dose filgotinib; filgotinib 200 mg QD, high-dose filgotinib; tofacitinib 5 mg BID, low-dose tofacitinib; tofacitinib 10 mg BID, high-dose tofacitinib; decernotinib 100 mg BID, low-dose decernotinib; decernotinib150 mg BID, high-dose decernotinib; baricitinib 2 mg QD, low-dose baricitinib; baricitinib 4 mg QD, high-dose baricitinib; upadacitinib 15 mg QD, low-dose upadacitinib; upadacitinib 30 mg QD, high-dose upadacitinib; peficitinib 100 mg QD, low-dose peficitinib; peficitinib 150 mg QD, high-dose peficitinib.

### Publication bias

Publication bias was tested using funnel plots for ACR20, ACR50, ACR70, and adverse events. In the funnel plots for ACR20, ACR50 and ACR70, some of the study points showed a slight bias distribution, suggesting that the results may have some publication bias. In the funnel plots for adverse events, the scattered distribution of each study was located inside the "funnel" and symmetrically distributed on both sides of the axis, indicating a lower likelihood of publishing bias (the funnel plots are shown in **S2 Fig**.

### Sensitivity analyses

Among the four research indicators, only the fixed-effects model and the random-effects model of ACR20 had a difference of DIC more than five (DIC of 194.41 and 177.19), and DIC of random-effects model of ACR20 was smaller; analyses were good for other indicators (**S3 Fig**).

## Discussion

Our review sought to evaluate the efficiency and safety of several JAK inhibitors in RA patients. A total of 33 RCTs with 15,961 participants were included in our study, and 12 interventions for the treatment of RA were thoroughly assessed. To our knowledge, this is the first study to use a Bayesian network meta-analysis to systematically evaluate the efficacy and safety of JAK inhibitors in the treatment of individuals with RA.

RA is a systemic immune system disease characterized by chronic arthritic inflammation and can lead to physical disability and reduced quality of life for patients [42]. JAK inhibitors are brand-new, small-molecule medications that are primarily used with other medications such as adenosine diphosphate and work primarily by competitively binding adenosine diphosphate to the structural domain of the kinase to produce their therapeutic effects [43]. Several RCTs in different countries have evaluated the efficacy of different JAK inhibitors as monotherapy in patients with RA by comparing them with placebo or ADA, however, we did not find any trials comparing JAK inhibitors directly against each other. Our network meta-analysis compared JAK inhibitors indirectly.

Our results imply that all JAK inhibitors were more effective than placebo when ACR20, ACR50, and ACR70 as efficacy indicators; specifically, high and low dose tofacitinib, low dose decernotinib, and high dose peficitinib were all more effective than adalimumab. Comparing JAK inhibitors against each other, decernotinib demonstrated the first in the SUCRA values of ACR50, ACR20and ACR70, suggesting the best efficacy of decernotinib among all the drugs. However, decernotinib showed the last in the SUCRA values of AE, suggesting that its safety is not satisfactory. At the same time, low-dose filgotinib was the least effective among all the JAK inhibitors ACR20/50/70.

Decernotinib as a relatively new JAK3 inhibitor [44] is not yet available in China. JAK3 inhibitors are found to be associated only with gamma chain receptor subunits that can participate in immune function and are identical to the receptors for IL-2, IL-4, IL-7, IL-9, IL-15, and IL-21, and not only that, the receptor subunits for these cytokines are associated with JAK1 inhibitor. This is why both JAK1 and JAK3 inhibitors can organize the signaling of transduction for these cytokines and thus alleviate symptoms in patients with RA [31]. However, while JAK3 inhibitors act, their selective inhibition does not affect the responses mediated by other JAK1 inhibitors (*e*.*g*. JAK-1/JAK-2, JAK-1/Tyk-2 and JAK-1/JAK-1) [45]. Therefore, the relationship between JAK3 inhibitors and γ-chain conduction was further studied later and may be helpful in elucidating the efficacy and safety of JAK3 inhibitors. Peficitinib, which is also a JAK3 inhibitor, acts in relation to its stereochemical mechanism, with a specific structure that determines its ability to bind to JAK3 molecules, inhibit JAK3 phosphorylation, and further block STAT phosphorylation thereby suppressing the synthesis of downstream inflammatory cytokine. Although the specific mechanism of action of peficitinib is unclear, it can block JAK3 and JAK1 to a large extent, JAK2 in small amounts, and rarely blocks TYK2. It mainly inhibits the signal transduction pathway of IFN-γ, not only having a slight inhibitory effect on IL-6, IL-12 and IL-23, but also exerting an inhibitory effect on Th1 cell differentiation and pathological Th17 cell proliferation. The above effects of peficitinib may be related to the adverse effects it produces [46].

In terms of the adverse events, expect high-dose baricitinib and upadacitinib, all the JAK inhibitors showed as safe as placebo. Comparing against each other, low-dose filgotinib is safer than high-dose upadacitinib, low-dose tofacitinib is safer than high-dose upadacitinib, and others showed no significant difference. The SUCRA of safety indicators revealed that low-dose filgotinib exhibited the best safety. Furthermore, this might be a result of the limited sample size used in the most recent JAK inhibitor research, the wide confidence interval, the lack of statistical differences in the results, and the lack of statistical efficacy, so the comparison of adverse events between JAK inhibitors requires further validation.

We should be cautious in interpreting the results of the meta only [47] because there are still shortcomings in this study: 1) The design methods of the 33 original studies included and the differences in the characteristics of the different populations may have some influences on the results of the study; 2) This study only included English literature and did not encompass literature in other languages, which may introduce some selection bias; 3) The safety of JAK inhibitors and other indicators such as CRP, ESR, and DAS-28 can be analyzed in the follow-up study, and their safety and other indicators should also be considered when JAK inhibitors with better efficacy are used; 4) JAK inhibitors are recently-developed drugs: head-to-head studies between certain JAK inhibitors have generated little or no direct evidence by which to judge the efficacy of different drug. The results obtained from the reticulated meta-analysis are indirectly comparable, which may cause some bias to the results of this study. To expound the efficiency and safety of medications, more direct RCTs are required.

In summary, the available evidence suggests significant efficacy with six different JAK inhibitors compared with placebo, with comparisons between different drugs at different doses, with decernotinib having the best efficacy and low-dose filgotinib having the best safety profile, but most of the differences between the safety profiles of JAK inhibition at different doses were not statistically significant. Although low-dose filgotinib ranked last among the efficacy indicators, it ranked first in safety indicators. Additionally, we could not neglect the higher risk of AEs with decernotinib as shown in the SUCRA results. Only the efficacy and safety of tofacitinib ranked higher among different SUCRA rankings. This suggests that low doses of tofacitinib can be used to treat RA given comprehensive consideration of efficacy and safety. Therefore, when we make decisions, we should take both efficiency and safety into account. Going forward more high-quality, multicenter, direct-controlled studies are expected to validate this study and to group JAK inhibitors according to their mechanism of action to see if there are differences in efficacy and safety between JAK inhibitors with different mechanisms of action.

## Supporting information

S1 Fig The results of the risk of bias evaluation. (TIF)

S2 Fig Funnel plots. (TIF)

S3 Fig Sensitivity analyses. (TIF)

S1 Table Study characteristics. (DOCX)

S1 File PRISMA 2009 checklist. (DOC)

S2 File Relevant code. (DOCX)

S3 File (ZIP)
