# Supplementary figures and images for: The efficacy and safety of different Janus kinase inhibitors as monotherapy in rheumatoid arthritis: A Bayesian network meta-analysis
Source: PLoS One. 2024 Jun 21;19(6):e0305621. doi: 10.1371/journal.pone.0305621 (PMC11192398; doi:10.1371/journal.pone.0305621)

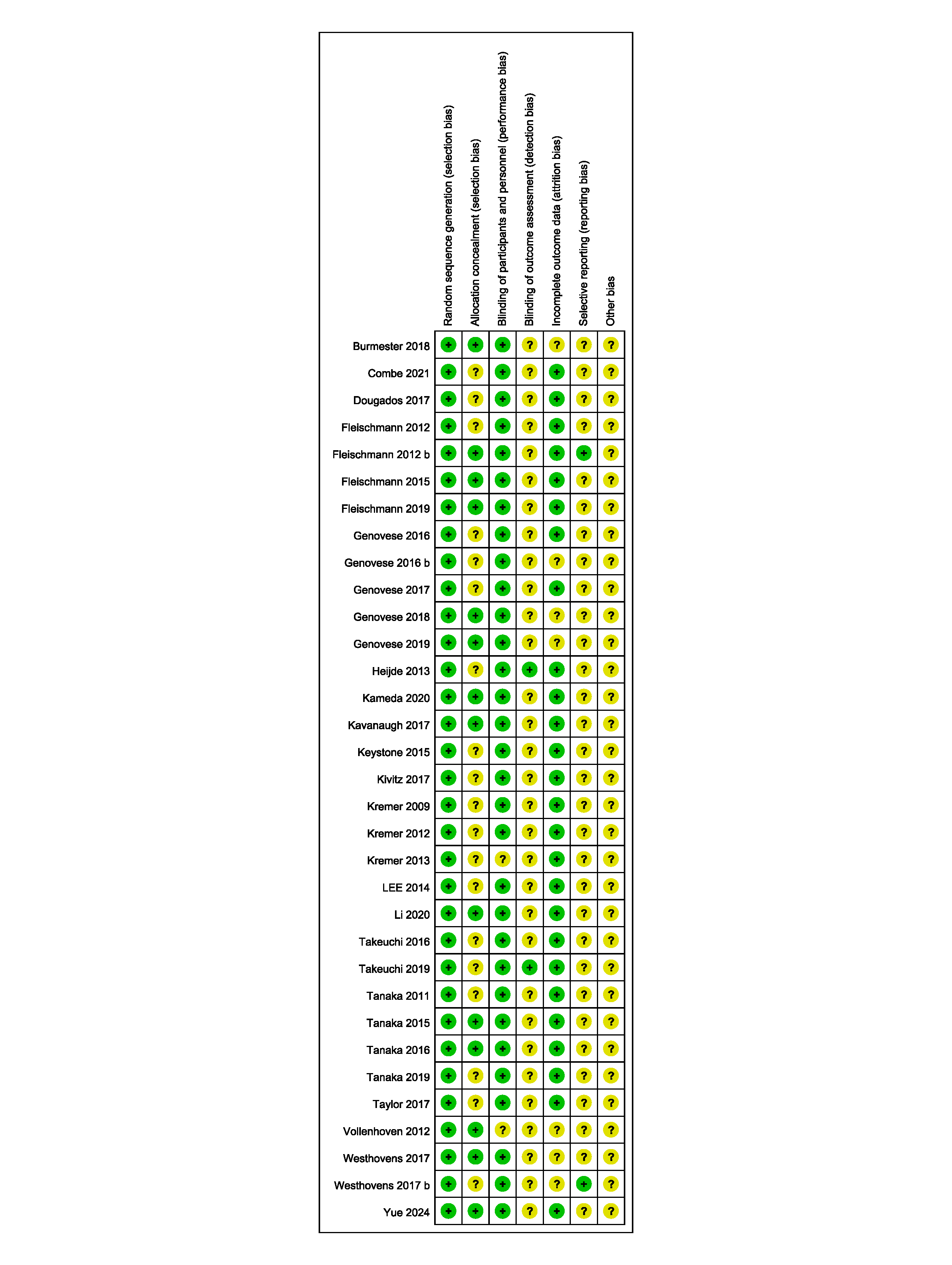

Supplement: S1 Fig — (TIF) [file pone.0305621.s001.tif]

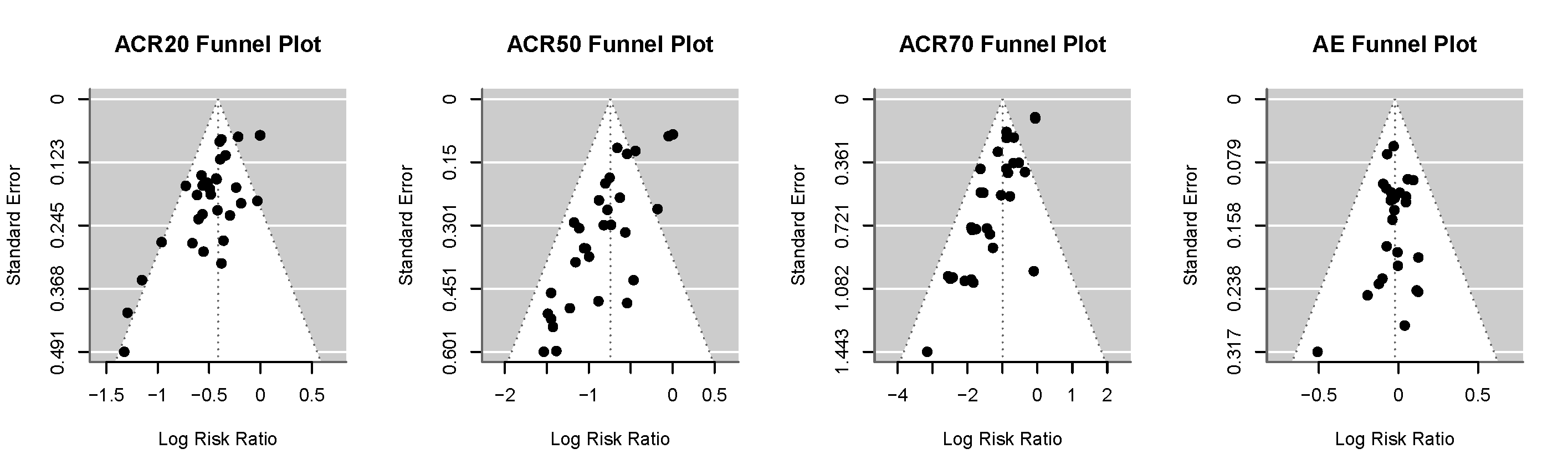

Supplement: S2 Fig — (TIF) [file pone.0305621.s002.tif]

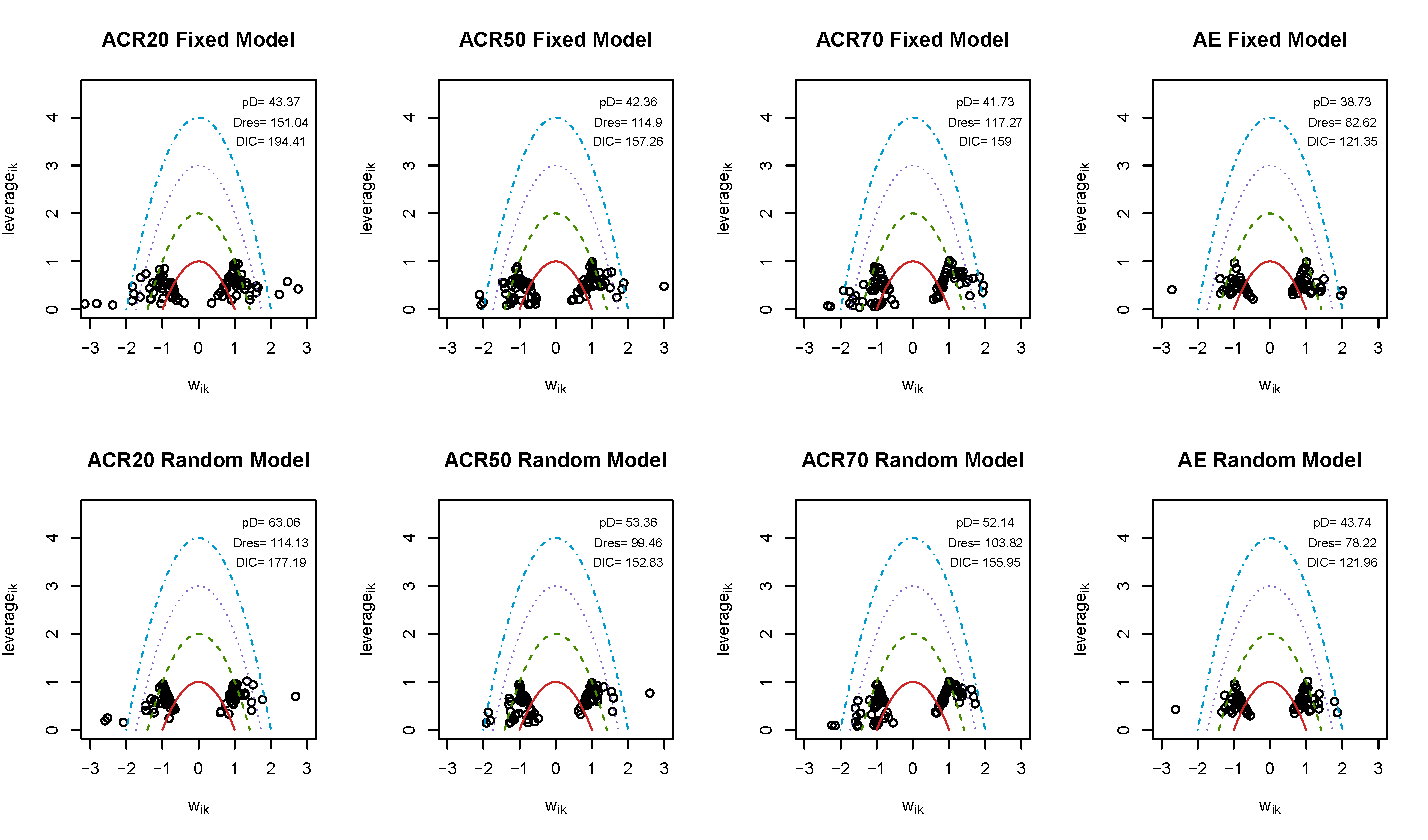

Supplement: S3 Fig — (TIF) [file pone.0305621.s003.tif]
