# Supplementary material for: The efficacy and safety of different Janus kinase inhibitors as monotherapy in rheumatoid arthritis: A Bayesian network meta-analysis
Source: PLoS One. 2024 Jun 21;19(6):e0305621. doi: 10.1371/journal.pone.0305621 (PMC11192398; doi:10.1371/journal.pone.0305621)
Supplement: S1 Table — (DOCX) [file pone.0305621.s004.docx]

**Table 1** Study details of all studies included in the

**S1 Table.** Study characteristics.

| **ID** | **Study** | **Phase** | **Background** | **Treatment name** | **Number** | **Age** | **Duration of disease** | **DAS28-ESR** | **DAS28-CRP** | **Time-point** | **Outcome** |
| --- | --- | --- | --- | --- | --- | --- | --- | --- | --- | --- | --- |
| **Tofacitinib** | |  |  |  |  |  |  |  |  |  |  |
| S1 | Fleischmann 2012 | Ⅱb | IR-DMARDs | tofacitinib 5mg BID | 49 | 54±13.5 | 8.1 | 6.6 | 5.6 | 12 weeks | ①②③ |
|  |  |  |  | tofacitinib 10 mg BID | 61 | 52±10.9 | 8.6 | 6.5 | 5.5 |  |  |
|  |  |  |  | ADA 40mg Q2W | 53 | 54±11.9 | 7.7 | 6.3 | 5.4 |  |  |
|  |  |  |  | placebo | 59 | 53±13.7 | 10.8 | 6.6 | 5.6 |  |  |
| S2 | Fleischmann 2012 b | Ⅲ | IR-DMARDs | tofacitinib 5mg BID | 243 | 52.2±11.5 | 8 | 6.71 | 5.68 | 3 months | ①②③④ |
|  |  |  |  | tofacitinib 10 mg BID | 245 | 52.4±11.7 | 8.6 | 6.7 | 5.6 |  |  |
|  |  |  |  | placebo | 122 | 49.7±12.4 | 7.7 | 6.65 | 5.56 |  |  |
| S3 | Kremer 2012 | Ⅱb | IR-MTX | tofacitinib 5mg BID | 71 | 52±12.8 | 9 | 6.1 | 5.1 | 12 weeks | ①②③ |
|  |  |  |  | tofacitinib 10 mg BID | 74 | 56±10.4 | 7.5 | 6.4 | 5.3 |  |  |
|  |  |  |  | placebo | 69 | 53±13.4 | 9.2 | 6.1 | 5.3 |  |  |
| S4 | Kremer 2009 | Ⅱa | IR-MTX | tofacitinib 5mg BID | 61 | 47.9±10.8 | 10.2 | NA | NA | 6 months | ①②③④ |
|  |  |  |  | placebo | 65 | 51.3±12.1 | 8.7 | NA | NA |  |  |
| S5 | Kremer 2013 |  | IR- DMARDs | tofacitinib 5mg BID | 315 | 52.7 | 8.1 | 6.27 | NA | 6 months | ④ |
|  |  |  |  | tofacitinib 10 mg BID | 318 | 51.9 | 9.2 | 6.36 | NA |  |  |
|  |  |  |  | placebo | 159 | NA | NA | NA | NA |  |  |
| S6 | Tanaka 2011 | Ⅱ | IR-MTX | tofacitinib 5mg BID | 27 | 50.0±9.8 | 8.3 | 6 | 5 | 12 weeks | ①②③④ |
|  |  |  |  | tofacitinib 10 mg BID | 26 | 50.6±10.0 | 7.1 | 5.9 | 4.9 |  |  |
|  |  |  |  | placebo | 28 | 50.6±12.4 | 8.4 | 5.9 | 4.9 |  |  |
| S7 | Tanaka 2015 | Ⅱ | IR-DMARDs | tofacitinib 5mg BID | 52 | 52.6 | 11 | 6.41 | NA | 12 weeks | ①②③④ |
|  |  |  |  | tofacitinib 10 mg BID | 53 | 54.7 | 7.3 | 6.06 | NA |  |  |
|  |  |  |  | placebo | 52 | 53.3 | 6.4 | 5.83 | NA |  |  |
| S8 | Vollenhoven 2012 | Ⅲ | MTX naive | tofacitinib 5mg BID | 204 | 53.0±11.9 | 7.6 | 6.6 | 5.4 | 3 months | ①②③④ |
|  |  |  |  | tofacitinib 10 mg BID | 201 | 52.9±11.8 | 7.4 | 6.5 | 5.4 |  |  |
|  |  |  |  | ADA 40mg Q2W | 204 | 52.5±11.7 | 8.1 | 6.4 | 5.3 |  |  |
|  |  |  |  | placebo | 108 | NA | NA | NA | NA |  |  |
| S9 | Heijde 2013 | Ⅲ | MTX naive | tofacitinib 5mg BID | 321 | 53.7±11.6 | 8.9 | 6.34 | 5.22 | 3 months | ④ |
|  |  |  |  | tofacitinib 10 mg BID | 316 | 52.0±11.4 | 9 | 6.25 | 5.2 |  |  |
|  |  |  |  | placebo | 160 | NA | NA | NA | NA |  |  |
| S10 | LEE 2014 | Ⅲ | MTX naïve | tofacitinib 5mg BID | 373 | 50.3 | 2.9 | 6.6 | NA | 24 weeks | ①②③④ |
|  |  |  |  | tofacitinib 10 mg BID | 397 | 49.3 | 3.4 | 6.5 | NA |  |  |
| **Baricitinib** | |  |  |  |  |  |  |  |  |  |  |
| S11 | Dougados 2017 | Ⅲ | IR-DMARDs | baricitinib 2mg QD | 229 | 52 | 8 | 6.3 | 5.6 | 12 weeks | ①②③④ |
|  |  |  |  | baricitinib 4mg QD | 227 | 52 | 8 | 6.2 | 5.6 |  |  |
|  |  |  |  | placebo | 228 | 51 | 7 | 6.2 | 5.5 |  |  |
| S12 | Genovese 2016 | Ⅱ | IR-TNFi,  IR-bioDMARDs | baricitinib 2mg QD | 174 | 55±11 | 14±8 | 6.7±1.0 | 6.0±0.9 | 12 weeks | ①②③④ |
|  |  |  |  | baricitinib 4mg QD | 177 | 56±11 | 14±9 | 6.6±1.1 | 5.9±1.0 |  |  |
|  |  |  |  | placebo | 176 | 56±11 | 14±10 | 6.6±0.9 | 5.9±0.9 |  |  |
| S13 | Keystone 2015 | Ⅱb | IR-MTX | baricitinib 2mg QD | 52 | 51±13 | 5.5±4.4 | 6.2±0.8 | 5.4±0.9 | 12 weeks | ①②③④ |
|  |  |  |  | baricitinib 4mg QD | 52 | 53±10 | 5.3±4.5 | 6.0±0.9 | 5.3±1.0 |  |  |
|  |  |  |  | placebo | 98 | 49±12 | 5.4±4.3 | 6.3±0.8 | 5.5±0.9 |  |  |
| S14 | Li 2020 | Ⅲ | IR-MTX | baricitinib 4mg QD | 145 | 49.5±10.6 | 10.7±8.3 | 6.6±1.0 | 5.9±1.0 | 12 weeks | ①②③④ |
|  |  |  |  | placebo | 145 | 48.9±12.7 | 9.1±7.0 | 6.7±0.9 | 6.0±0.9 |  |  |
| S15 | Tanaka 2016 | Ⅱb | IR-MTX | baricitinib 2mg QD | 24 | 56.1 | 6.32 | 5.78 | 4.94 | 12 weeks | ①②③④ |
|  |  |  |  | baricitinib 4mg QD | 24 | 57.5 | 5.86 | 5.77 | 4.96 |  |  |
|  |  |  |  | placebo | 49 | 51.1 | 5.06 | 5.53 | 4.72 |  |  |
| S16 | Taylor 2017 | Ⅲ | MTX naive | baricitinib 4mg QD | 487 | 54±2 | 10±9 | 6.5±0.9 | 5.8±0.9 | 24 weeks | ①②③④ |
|  |  |  |  | ADA 40mg Q2W | 330 | 53±12 | 10±9 | 6.4±1.0 | 5.8±0.9 |  |  |
|  |  |  |  | placebo | 488 | 53±2 | 10±9 | 6.4±1.0 | 5.7±1.0 |  |  |
| **Peficitinib** | |  |  |  |  |  |  |  |  |  |  |
| S17 | Genovese 2017 | Ⅱb | IR-csDMARDs | peficitinib 100mg QD | 58 | 54.9±11.3 | 11.0±8.4 | 6.6±0.8 | 5.7±0.9 | 12 weeks | ①②③④ |
|  |  |  |  | peficitinib 150mg QD | 64 | 54.4±12.5 | 10.5±8.2 | 6.7±1.0 | 5.9±1.1 |  |  |
|  |  |  |  | placebo | 51 | 52.7±12.2 | 9.8±7.7 | 6.6±0.9 | 5.9±0.9 |  |  |
| S18 | Kivitz 2017 | Ⅱb | IR-MTX | peficitinib 100mg QD | 84 | 54.5±12.8 | 7.5±7.8 | 6.4±0.9 | 5.6±1.0 | 12 weeks | ①②③④ |
|  |  |  |  | peficitinib 150mg QD | 78 | 54.2±12.5 | 7.3±6.3 | 6.4±0.9 | 5.6±0.9 |  |  |
|  |  |  |  | placebo | 72 | 52.6±12.2 | 7.2±7.7 | 6.2±0.8 | 5.4±0.9 |  |  |
| S19 | Takeuchi 2016 | Ⅱb |  | peficitinib 100mg QD | 55 | 52.1±12.1 | 7.58±7.12 | 6.07±1.02 | 5.34±1.07 | 12 weeks | ①②③④ |
|  |  |  |  | peficitinib 150mg QD | 58 | 51.6±12.1 | 6.95±5.17 | 6.08±1.07 | 5.41±1.12 |  |  |
|  |  |  |  | placebo | 56 | 54.2±12.1 | 6.92±5.39 | 5.77±0.91 | 5.10±1.01 |  |  |
| S20 | Takeuchi 2019 | Ⅲ | IR-MTX | peficitinib 100mg QD | 174 | 58.5 | 4.41 | 5.83 | 5.21 | 12 weeks | ①②③④ |
|  |  |  |  | peficitinib 150mg QD | 174 | 56.2 | 4.37 | 5.98 | 5.36 |  |  |
|  |  |  |  | placebo | 170 | 55.3 | 4.3 | 6.05 | 5.41 |  |  |
| S21 | Tanaka 2019 | Ⅲ | IR-DMARDs | peficitinib 100mg QD | 104 | 54.1 | 8.75 | 5.94 | 5.29 | 12 weeks | ①②③④ |
|  |  |  |  | peficitinib 150mg QD | 102 | 55 | 10.39 | 6.01 | 5.41 |  |  |
|  |  |  |  | placebo | 101 | 56.3 | 6.98 | 6.03 | 5.43 |  |  |
| S22 | Yue 2024 | Ⅲ | IR-MTX | peficitinib 100mg QD | 129 | 50.9±11.7 | 5.7±6.2 | 6.2±1.0 | 5.8±1.0 | 24 weeks | ①④ |
|  |  |  |  | peficitinib 150mg QD | 128 | 48.9±11.2 | 6.2±6.5 | 6.3±1.1 | 5.7±1.0 |  |  |
|  |  |  |  | placebo | 128 | 50.2±10.9 | 5.9±7.0 | 6.3±1.0 | 5.7±0.9 |  |  |
| **Decernotinib** | |  |  |  |  |  |  |  |  |  |  |
| S23 | Fleischmann 2015 | Ⅱa | IR-DMARDs | decernotinib 100mg BID | 40 | 56.5±8.9 | 6.7±6.2 | NA | 6.0±0.9 | 12 weeks | ①②③④ |
|  |  |  |  | decernotinib 150mg BID | 41 | 57.0±9.3 | 7.1±7.6 | NA | 6.1±0.9 |  |  |
|  |  |  |  | placebo | 41 | 54.9±10.6 | 10.0±9.6 | NA | 6.0±1.0 |  |  |
| S24 | Genovese 2016 | Ⅱb | IR-MTX | decernotinib 100mg BID | 72 | 55.7±12.2 | 7.7±7.5 | 6.29±0.818 | 5.7±0.8 | 12 weeks | ①②③ |
|  |  |  |  | placebo | 71 | 52.7±13.2 | 7.2±7.5 | 6.46±0.764 | 5.8±0.8 |  |  |
| **Filgotinib** | |  |  |  |  |  |  |  |  |  |  |
| S25 | Genovese 2019 | Ⅲ | IR-bDMARDs | filgotinib 100mg QD | 153 | 55 | 10.3 | NA | 5.9 | 12 weeks | ①②③④ |
|  |  |  |  | filgotinib 200mg QD | 147 | 56 | 9.8 | NA | 5.9 |  |  |
|  |  |  |  | placebo | 148 | 56 | 9.9 | NA | 5.9 |  |  |
| S26 | Kavanaugh 2017 | Ⅱb | IR-MTX | filgotinib 100mg QD | 70 | 53 | 9 | NA | 6.18 | 12 weeks | ①②③④ |
|  |  |  |  | filgotinib 200mg QD | 69 | 52 | 9 | NA | 6.09 |  |  |
|  |  |  |  | placebo | 72 | 52 | 10 | NA | 6.22 |  |  |
| S27 | Westhovens 2017 | Ⅱb | IR-MTX | filgotinib 100mg QD | 85 | 52 | 8 | NA | 6.14 | 12 weeks | ①②③ |
|  |  |  |  | filgotinib 200mg QD | 86 | 55 | 9 | NA | 6.22 |  |  |
|  |  |  |  | placebo | 86 | 52 | 8 | NA | 5.98 |  |  |
| S28 | Combe 2021 | Ⅲ | IR-MTX | filgotinib 100mg QD | 480 | 53±12.6 | 8.5±8.2 | NA | 5.7±1.0 | 12 weeks | ①②③ |
|  |  |  |  | filgotinib 200mg QD | 475 | 52±12.8 | 7.3±7.4 | NA | 5.8±0.9 |  |  |
|  |  |  |  | ADA 40mg Q2W | 325 | 53±12.9 | 8.0±7.4 | NA | 5.7±0.9 |  |  |
|  |  |  |  | placebo | 475 | 53±12.8 | 7.3±7.2 | NA | 5.7±0.9 |  |  |
| S29 | Westhovens 2017 b | Ⅲ | IR-MTX | filgotinib 100mg QD | 207 | 54±12.6 | 2.3±4.7 | NA | 5.7±1.0 | 24 weeks | ①②③ |
|  |  |  |  | filgotinib 200mg QD | 416 | 53±13.8 | 1.9±3.6 | NA | 5.7±1.0 |  |  |
| **Upadacitinib** | |  |  |  |  |  |  |  |  |  |  |
|  |  |  |  |  |  |  |  |  |  |  |  |
| S30 | Burmester 2018 | Ⅲ | IR-csDMARDs | upadacitinib 15mg QD | 221 | 55.3 | 7.3 | NA | 5.7 | 12 weeks | ①②③④ |
|  |  |  |  | upadacitinib 30mg QD | 219 | 55.8 | 7.3 | NA | 5.7 |  |  |
|  |  |  |  | placebo | 221 | 56 | 7.2 | NA | 5.6 |  |  |
| S31 | Fleischmann 2019 | Ⅲ | IR-MTX | upadacitinib 15mg QD | 651 | 54±12 | 8±8 | 6.4±1.0 | 5.8±1.0 | 12 weeks | ①②③ |
|  |  |  |  | ADA 40mg Q2W | 327 | 54±12 | 8±8 | 6.5±1.0 | 5.9±1.0 |  |  |
|  |  |  |  | placebo | 651 | 54±12 | 8±8 | 6.5±1.0 | 5.8±0.9 |  |  |
| S32 | Genovese 2018 | Ⅲ | IR-csDMARDs | upadacitinib 15mg QD | 164 | 56.3 | 12.4 | NA | NA | 12 weeks | ①②③④ |
|  |  |  |  | upadacitinib 30mg QD | 165 | 57.3 | 12.7 | NA | NA |  |  |
|  |  |  |  | placebo | 169 | 57.6 | 14.5 | NA | NA |  |  |
| S33 | Kameda 2020 | Ⅱb/Ⅲ | IR-csDMARDs | upadacitinib 15mg QD | 49 | 56 | 2.9 | NA | 5.1 | 12 weeks | ①②③④ |
|  |  |  |  | upadacitinib 30mg QD | 50 | 54.7 | 2.8 | NA | 5 |  |  |
|  |  |  |  | placebo | 49 | 54.3 | 2.1 | NA | 5.2 |  |  |

DMARDs: Disease-modifying anti-rheumatic drugs, IR: Inadequate Response, MTX: Methotrexate, bio: Biologic, DAS: Disease Activity Score; ①: ACR20, ②:ACR50, ③: ACR70, ④: Adverse events.

**Figure S3.** Sensitivity Analyses.

indicators
