# Supplementary material for: The efficacy and safety of different Janus kinase inhibitors as monotherapy in rheumatoid arthritis: A Bayesian network meta-analysis
Source: PLoS One. 2024 Jun 21;19(6):e0305621. doi: 10.1371/journal.pone.0305621 (PMC11192398; doi:10.1371/journal.pone.0305621)
Supplement: S2 File — (DOCX) [file pone.0305621.s006.docx]

install.packages("rjags")

install.packages("metafor")

install.packages("netmeta")

install.packages("gemtc")

install.packages("ggplot2")

install.packages("pkgbuild")

install.packages("BUGSnet")

install.packages("rmarkdown")

install.packages("xlsx")

install.packages("rJava")

install.packages("rJava",type="binary")

library("pkgbuild")

pkgbuild::has_build_tools()

install.packages(c("remotes","knitr"))

remotes::install_github("audrey-b/BUGSnet@v1.1.0",upgrad=TRUE,build_vignettes = TRUE,dependencies = TRUE)

install.packages(c("xlsx","dplyr","tidyr"))

library(xlsx)

library(dplyr)

library(tidyr)

library(ggplot2)

library(BUGSnet)

library(rjags)

library(gemtc)

library(metafor)

library(netmeta)

library(Matrix)

library(mutate)

library(meta)

usethis::edit_r_environ()

setwd("d:/毕业论文新数据/renewR")

setwd("d:/JAK meta投稿相关/更改正文和补充")

data_ACR20<-read.csv("JAKACR20.csv",sep = ",",header = T)

data_ACR50<-read.csv("JAKACR50.csv",sep=",",header = T)

data_ACR70<-read.csv("JAKACR70.csv",sep = ",",header = T)

data_AE<-read.csv("JAKAE.csv",sep=",",header = T)

#构建网络

data_ACR20<-data.prep(arm.data =data_ACR20,varname.t = "treatment",varname.s = "study" )

data_ACR50<-data.prep(arm.data = data_ACR50,varname.t = "treatment",varname.s = "study")

data_ACR70<-data.prep(arm.data = data_ACR70,varname.t = "treatment",varname.s = "study")

data_AE<-data.prep(arm.data=data_AE,varname.t = "treatment",varname.s = "study")

par(mfrow = c(1,1))

n1<-net.plot(data_ACR20,node.scale = 2.45,node.lab.cex= 0.74)

n2<-net.plot(data_ACR50,node.scale = 2.45,node.lab.cex= 0.74)

n3<-net.plot(data_ACR70,node.scale = 2.45,node.lab.cex= 0.74)

n4<-net.plot(data_AE,node.scale = 2.45,node.lab.cex= 0.74)

tiff("JAKACR20网状图.tiff", height=8000,width=4000, res= 600)

net.plot(data_ACR20)

dev.off()

tiff("JAKACR50网状图.tiff", height=8000,width=4000, res= 600)

net.plot(data_ACR50)

dev.off()

tiff("JAKACR70网状图.tiff", height=8000,width=4000, res= 600)

net.plot(data_ACR70)

dev.off()

tiff("JAKAE网状图.tiff", height=8000,width=4000, res= 600)

net.plot(data_AE)

dev.off()

fixed_effects_model_ACR20<-nma.model(data_ACR20,outcome = "responders",N="sampleSize",reference = "Placebo",family = "binomial",link = "log",effects = "fixed")

fixed_effects_model_ACR50<-nma.model(data_ACR50,outcome = "responders",N="sampleSize",reference = "Placebo",family = "binomial",link = "log",effects = "fixed")

fixed_effects_model_ACR70<-nma.model(data_ACR70,outcome = "responders",N="sampleSize",reference = "Placebo",family = "binomial",link = "log",effects = "fixed")

fixed_effects_model_AE<-nma.model(data_AE,outcome = "responders",N="sampleSize",reference = "Placebo",family = "binomial",link = "log",effects = "fixed")

random_effects_model_ACR20<-nma.model(data_ACR20,outcome = "responders",N="sampleSize",reference = "Placebo",family = "binomial",link = "log",effects = "random")

random_effects_model_ACR50<-nma.model(data_ACR50,outcome = "responders",N="sampleSize",reference = "Placebo",family = "binomial",link = "log",effects = "random")

random_effects_model_ACR70<-nma.model(data_ACR70,outcome = "responders",N="sampleSize",reference = "Placebo",family = "binomial",link = "log",effects = "random")

random_effects_model_AE<-nma.model(data_AE,outcome = "responders",N="sampleSize",reference = "Placebo",family = "binomial",link = "log",effects = "random")

inconsistency_model_ACR20<-nma.model(data_ACR20,outcome = "responders",N="sampleSize",reference = "Placebo",family = "binomial",link = "log",type = "inconsistency",effects = "random")

inconsistency_model_ACR50<-nma.model(data_ACR50,outcome = "responders",N="sampleSize",reference = "Placebo",family = "binomial",link = "log",type = "inconsistency",effects = "random")

inconsistency_model_ACR70<-nma.model(data_ACR70,outcome = "responders",N="sampleSize",reference = "Placebo",family = "binomial",link = "log",type = "inconsistency",effects = "random")

inconsistency_model_AE<-nma.model(data_AE,outcome = "responders",N="sampleSize",reference = "Placebo",family = "binomial",link = "log",type = "inconsistency",effects = "random")

consistency_model_ACR20<-nma.model(data_ACR20,outcome = "responders",N="sampleSize",reference = "Placebo",family = "binomial",link = "log",type = "consistency",effects = "random")

consistency_model_ACR50<-nma.model(data_ACR50,outcome = "responders",N="sampleSize",reference = "Placebo",family = "binomial",link = "log",type = "consistency",effects = "random")

consistency_model_ACR70<-nma.model(data_ACR70,outcome = "responders",N="sampleSize",reference = "Placebo",family = "binomial",link = "log",type = "consistency",effects = "random")

consistency_model_AE<-nma.model(data_AE,outcome = "responders",N="sampleSize",reference = "Placebo",family = "binomial",link = "log",type = "consistency",effects = "random")

fixed_effects_results_ACR20<-nma.run(fixed_effects_model_ACR20,n.adapt = 10000,n.iter = 50000)

fixed_effects_results_ACR50<-nma.run(fixed_effects_model_ACR50,n.adapt = 10000,n.iter = 50000)

fixed_effects_results_ACR70<-nma.run(fixed_effects_model_ACR70,n.adapt = 10000,n.iter = 50000)

fixed_effects_results_AE<-nma.run(fixed_effects_model_AE,n.adapt = 10000,n.iter = 50000)

random_effects_results_ACR20<-nma.run(random_effects_model_ACR20,n.adapt=10000,n.iter = 50000)

random_effects_results_ACR50<-nma.run(random_effects_model_ACR50,n.adapt=10000,n.iter = 50000)

random_effects_results_ACR70<-nma.run(random_effects_model_ACR70,n.adapt=10000,n.iter = 50000)

random_effects_results_AE<-nma.run(random_effects_model_AE,n.adapt=10000,n.iter = 50000)

inconsistency_result_ACR20<-nma.run(inconsistency_model_ACR20,n.adapt = 10000,n.iter = 50000)

inconsistency_result_ACR50<-nma.run(inconsistency_model_ACR50,n.adapt = 10000,n.iter = 50000)

inconsistency_result_ACR70<-nma.run(inconsistency_model_ACR70,n.adapt = 10000,n.iter = 50000)

inconsistency_result_AE<-nma.run(inconsistency_model_AE,n.adapt = 10000,n.iter = 50000)

consistency_result_ACR20<-nma.run(consistency_model_ACR20,n.adapt = 10000,n.iter = 50000)

consistency_result_ACR50<-nma.run(consistency_model_ACR50,n.adapt = 10000,n.iter = 50000)

consistency_result_ACR70<-nma.run(consistency_model_ACR70,n.adapt = 10000,n.iter = 50000)

consistency_result_AE<-nma.run(consistency_model_AE,n.adapt = 10000,n.iter = 50000)

par(mfrow=c(2,4))

nma.fit(fixed_effects_results_ACR20,main = "ACR20 Fixed Model")

nma.fit(fixed_effects_results_ACR50,main = "ACR50 Fixed Model")

nma.fit(fixed_effects_results_ACR70,main = "ACR70 Fixed Model")

nma.fit(random_effects_results_ACR20,main = "ACR20 Random Model")

nma.fit(random_effects_results_ACR50,main = "ACR50 Random Model")

nma.fit(random_effects_results_ACR70,main = "ACR70 Random Model")

par(mfrow=c(2,2))

nma.fit(fixed_effects_results_AE,main = "AE Fixed Model")

nma.fit(random_effects_results_AE,main = "AE Random Model")

par(mfrow=c(2,4))

nma.fit(inconsistency_result_ACR20,main = "ACR20 Inconsistency Model")

nma.fit(inconsistency_result_ACR50,main = "ACR50 Inconsistency Model")

nma.fit(inconsistency_result_ACR70,main = "ACR70 Inconsistency Model")

nma.fit(consistency_result_ACR20,main = "ACR20 Consistency Model")

nma.fit(consistency_result_ACR50,main = "ACR50 Consistency Model")

nma.fit(consistency_result_ACR70,main = "ACR70 Consistency Model")

nma.fit(inconsistency_result_AE,main = "AE Inconsistency Model")

nma.fit(consistency_result_AE,main = "AE Consistency Model")

memory.size(F)

memory.limit()

object.size()

memory.limit(15010)

#计算sucra值#

sucra_out_ACR20<-nma.rank(consistency_result_ACR20,largerbetter = TRUE)

sucra_out_ACR50<-nma.rank(consistency_result_ACR50,largerbetter = TRUE)

sucra_out_ACR70<-nma.rank(consistency_result_ACR70,largerbetter = TRUE)

sucra_out_AE<-nma.rank(consistency_result_AE,largerbetter = FALSE)

#sucra折图

s1<-sucra_out_ACR20$sucraplot

s2<-sucra_out_ACR50$sucraplot

s3<-sucra_out_ACR70$sucraplot

s4<-sucra_out_AE$sucraplot

#累计面积概率图

r1<-sucra_out_ACR20$rankogram+theme(axis.text.x = element_text(angle = 30,hjust = 1,vjust = 1))

r2<-sucra_out_ACR50$rankogram+theme(axis.text.x = element_text(angle = 30,hjust = 1,vjust = 1))

r3<-sucra_out_ACR70$rankogram+theme(axis.text.x = element_text(angle = 30,hjust = 1,vjust = 1))

library(gridExtra)

grid.arrange(s1,s2,s3,r1,r2,r3,ncol=3)

r4<-sucra_out_AE$rankogram+theme(axis.text.x = element_text(angle = 30,hjust = 1,vjust = 1))

grid.arrange(s4,s5,r4,r5,ncol=2)

grid.arrange(s1,r1,ncol=2)

grid.arrange(s2,r2,ncol=2)

grid.arrange(s3,r3,ncol=2)

grid.arrange(s4,r4,ncol=2)

# sucra #

ss1<-sucra_out_ACR20$sucratable

ss2<-sucra_out_ACR50$sucratable

ss3<-sucra_out_ACR70$sucratable

grid.arrange(ss1,ss2,ss3,ncol=3)

ss1

ss2

ss3

ss4<-sucra_out_AE$sucratable

ss4

write.csv(ss1,"ACR20-sucra.csv")

write.csv(ss2,"ACR50-sucra.csv")

write.csv(ss3,"ACR70-sucra.csv")

write.csv(ss4,"AE-sucra.csv")

#联赛热图

league.out_ACR20<-nma.league(consistency_result_ACR20,central.tdcy = "median",order = sucra_out_ACR20$order,log.scale = FALSE,low.colour = "springgreen4",mid.colour = "white",high.colour = "red",digits = 2)

league.out_ACR50<-nma.league(consistency_result_ACR50,central.tdcy = "median",order = sucra_out_ACR50$order,log.scale = FALSE,low.colour = "springgreen4",mid.colour = "white",high.colour = "red",digits = 2)

league.out_ACR70<-nma.league(consistency_result_ACR70,central.tdcy = "median",order = sucra_out_ACR70$order,log.scale = FALSE,low.colour = "springgreen4",mid.colour = "white",high.colour = "red",digits = 2)

league.out_AE<-nma.league(consistency_result_AE,central.tdcy = "median",order = sucra_out_AE$order,log.scale = FALSE,low.colour = "springgreen4",mid.colour = "white",high.colour = "red",digits = 2)

l1<-league.out_ACR20$heatplot

l2<-league.out_ACR50$heatplot

l3<-league.out_ACR70$heatplot

grid.arrange(l1,l2,l3,ncol=1)

l4<-league.out_AE$heatplot

l1

l2

l3

l4
